# A one-week reduced-carbohydrate diet to mitigate iatrogenic peripheral hyperinsulinemia does not improve insulin sensitivity or endothelial function in a randomized, crossover trial in patients with type 1 diabetes

**DOI:** 10.1186/s12933-025-02658-z

**Published:** 2025-03-05

**Authors:** Justin M. Gregory, T. Jordan Smith, Sara H. Duffus, David Brooks, M. Naweed Akbar, Margaret-Anne Huntley, JoAnn A. Gottlieb, Lauren M. LeStourgeon, Christopher S. Wilson, Joshua A. Beckman, Alan D. Cherrington

**Affiliations:** 1 Ian Burr Division of Pediatric Endocrinology and Diabetes, Vanderbilt University School of Medicine, 1500 21st Avenue South, Suite 1514, Nashville, TN 37212-3157 USA; 2 Division of Pediatric Endocrinology, University of North Carolina, 127 Medical School Wing E, CB# 7039, Chapel Hill, NC 27599-7039 USA; 3 Mildred Stahlman Division of Neonatology, Vanderbilt University School of Medicine, 1161 21st Ave S A0126, Nashville, TN 37232 USA; 4 Department of Pediatrics, Creighton University, 2412 Cuming St #103, Omaha, NE 68131 USA; 5 Vanderbilt Institute for Clinical and Translational Research, Vanderbilt University School of Medicine, 2525 West End Ave., Nashville, TN 37203-8820 USA; 6 Department of Internal Medicine, Vanderbilt University School of Medicine, 1161 21st Ave S, Nashville, TN 37232 USA; 7 Division of Vascular Medicine, University of Texas Southwestern, 5323 Harry Hines Blvd., Dallas, TX 75390 USA; 8 Department of Molecular Physiology and Biophysics, Vanderbilt University School of Medicine, 2301 Vanderbilt Place, Nashville, TN 37240 USA

**Keywords:** Endothelial dysfunction, Endothelial function, Hyperinsulinemia, Insulin resistance, Insulin sensitivity, Reduced-carbohydrate diet, Type 1 diabetes

## Abstract

**Background:**

Iatrogenic peripheral hyperinsulinemia, resulting from peripheral insulin administration in type 1 diabetes, may increase insulin resistance and impair endothelial function. We hypothesized that lowering iatrogenic peripheral hyperinsulinemia via a one-week, reduced-carbohydrate diet (RCD) would improve insulin sensitivity and endothelial function compared with an isocaloric standard carbohydrate diet (SCD).

**Methods:**

In this randomized, single-blinded, crossover trial, we studied 12 adults with type 1 diabetes. Participants completed both a one-week RCD and a one-week SCD, separated by a three-week washout. After each intervention, we measured insulin sensitivity using a hyperinsulinemic–euglycemic clamp and assessed endothelial function via brachial-artery flow-mediated vasodilation (FMD).

**Results:**

The RCD reduced total daily insulin doses by 16% compared with the SCD. Despite this reduction, insulin sensitivity did not improve (median glucose infusion rates: RCD 8.1 mg/kg FFM/min [IQR 6.7–10.1] vs. SCD 8.6 mg/kg FFM/min [7.0–11.0], *p* = 0.47). Similarly, endothelial function did not differ significantly (FMD after RCD 7.50% [3.25–15.5] vs. SCD 9.81% [4.96–14.3], *p* = 0.91). Although higher insulin doses correlated with lower insulin sensitivity under both conditions, lowering insulin dose through the RCD alone did not yield measurable improvements.

**Conclusions:**

Although a one-week RCD significantly lowered insulin requirements, it failed to enhance insulin sensitivity or endothelial function in adults with type 1 diabetes. These findings underscore the complex and dynamic relationship between insulin exposure and cardiometabolic health. Similar basal overnight insulin delivery may have masked potential benefits by the time of testing, highlighting the need for further studies to refine strategies aimed at mitigating hyperinsulinemia’s adverse effects.

**Trial registration:**

ClinicalTrials.gov NCT04118374.

**Graphical abstract:**

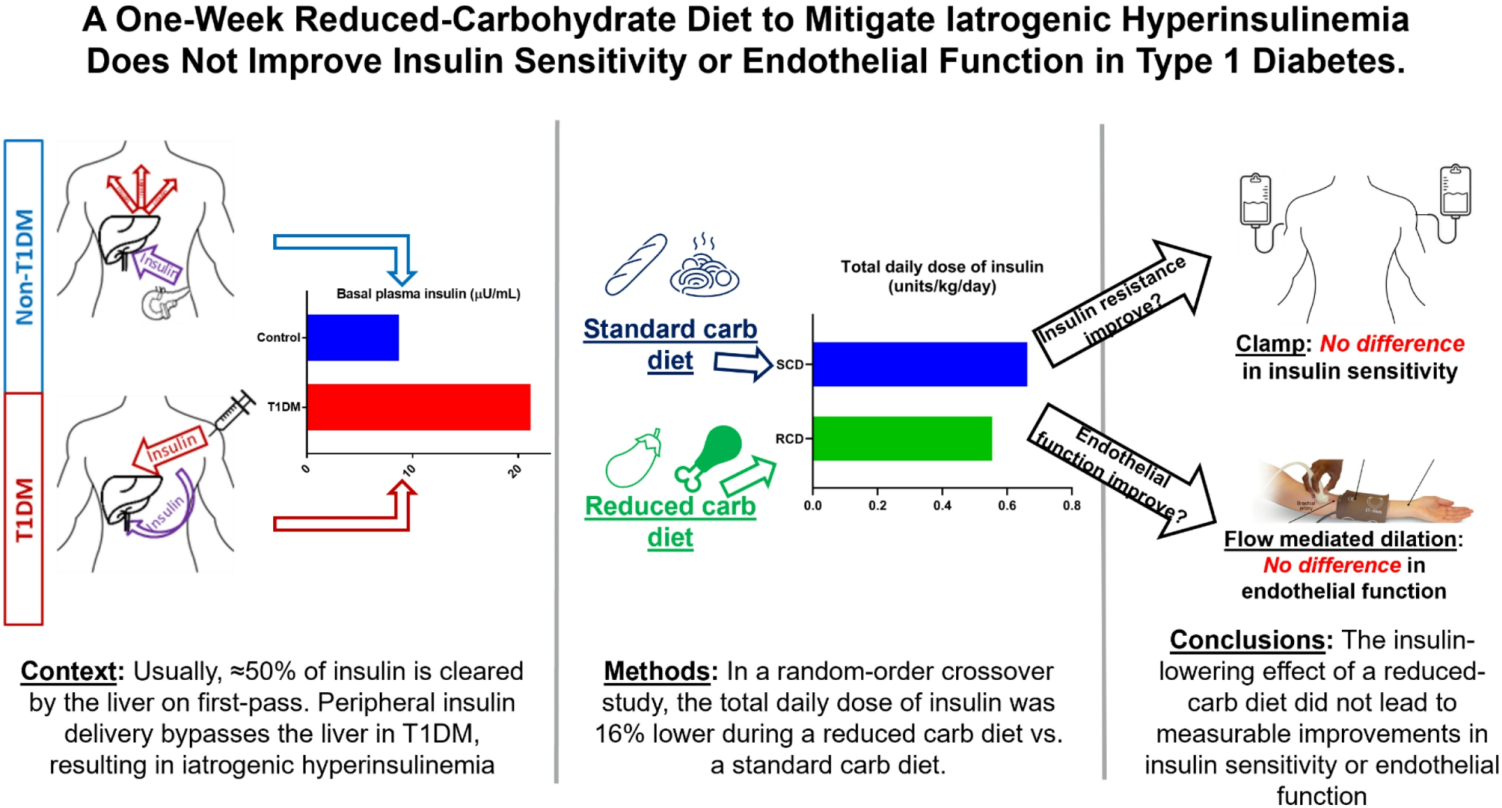

**Supplementary Information:**

The online version contains supplementary material available at 10.1186/s12933-025-02658-z.

## Introduction

Macrovascular disease remains the leading cause of mortality in type 1 diabetes, persisting despite intense efforts to reduce hyperglycemia since the Diabetes Control and Complications Trial [1–4]. Adolescents with type 1 diabetes may experience as much as a 13-year reduction in life expectancy, predominantly due to ischemic heart disease [4]. Alarmingly, this risk persists even in patients achieving target glycemic control, defined as an A1c ≤ 6.9% (52 mmol/mol) [2]. The roots of macrovascular disease extend into childhood and adolescence [5–8], highlighting the need for new long-term strategies beyond glycemic management to mitigate this risk in type 1 diabetes.

In the complex landscape of macrovascular disease of type 1 diabetes, insulin resistance and endothelial dysfunction emerge as key factors, closely intertwined with cardiovascular risk. The conventional method of subcutaneous insulin administration delivers insulin directly into the peripheral circulation—bypassing first-pass hepatic clearance–and thereby results in iatrogenic peripheral circulation hyperinsulinemia. Studies in healthy individuals have shown that sustained insulin levels in the range of approximately 120–180 pmol/L (20–30 µU/mL) can diminish insulin sensitivity [9–11] and endothelial function [12], independent of glycemia. Notably, these insulin concentrations are similar to basal levels observed in type 1 diabetes patients during peripheral insulin delivery [13–15]. Given the close links between insulin resistance, endothelial dysfunction, and cardiovascular disease, addressing the dual impact of iatrogenic peripheral hyperinsulinemia on insulin sensitivity [16] and endothelial health [17] becomes an important target for reducing the burden of cardiovascular disease in type 1 diabetes.

Current therapeutic strategies to reduce iatrogenic peripheral hyperinsulinemia in type 1 diabetes are limited and often carry significant shortcomings. Although hepatopreferential and oral insulin analogs [18, 19], as well as intraperitoneal insulin delivery [20], show promise in preclinical studies, none are currently available for clinical use [21–23]. Adjunctive therapies such as SGLT2 inhibitors and GLP-1 receptor agonists can lower hyperinsulinemia but pose risks like diabetic ketoacidosis [24] or gastrointestinal side effects [25], limiting their utility. Furthermore, these interventions have only led to modest reductions in insulin dosage and small improvements in glycemic control [25–29]. This situation highlights a significant shortfall in addressing iatrogenic peripheral hyperinsulinemia without excessive adverse effects. Against this backdrop, a reduced-carbohydrate diet (RCD) emerges as a viable non-pharmacologic strategy. Because prandial insulin is often dosed proportionally to carbohydrate intake in type 1 diabetes, the RCD offers a means to decrease the total daily dose of insulin (TDD_insulin_), potentially mitigating insulin resistance and endothelial dysfunction associated with iatrogenic peripheral hyperinsulinemia. This approach aligns with the goal of minimizing exogenous insulin use and offers a non-pharmacological option to address cardiovascular risk in type 1 diabetes.

Given the potential role of iatrogenic peripheral hyperinsulinemia in worsening insulin resistance and endothelial dysfunction, we hypothesized that reducing this hyperinsulinemia via an RCD would improve insulin sensitivity and endothelial function in individuals with type 1 diabetes. To test this hypothesis, we conducted a single-blinded, random-order crossover study comparing the effects of a one-week RCD to an isocaloric, standard carbohydrate diet (SCD) on insulin sensitivity and endothelial function in adults with type 1 diabetes.

## Methods

### Participants

We recruited adults with type 1 diabetes (age 18–60 years, BMI 18–33 kg/m^2^, HbA1c 5.9–9.0%) from the Vanderbilt Eskind Diabetes Clinic and community sources. Key exclusion criteria were recent severe hypoglycemia within the past three months, diabetic ketoacidosis within the past six months, current pregnancy, anemia, and medications affecting insulin sensitivity or endothelial function (see Supplemental Table 1 for complete criteria). Enrollment began in November 2021 and concluded by March 2024.

### Study design

We conducted a single-blinded, random-order crossover study to compare the effects of the RCD versus SCD on insulin sensitivity (primary outcome) and endothelial function (key secondary outcome). We chose a crossover design to reduce inter-individual variability by allowing each participant to serve as their own control. Participants completed three visits at Vanderbilt’s Clinical Research Center: an initial screening then two research visits to determine the study outcomes after each nutritional intervention.

#### Screening visit

After obtaining written informed consent, we determined eligibility through a physical exam and laboratory tests for A1c, hematocrit, hepatic transaminases, pregnancy status, and estimated glomerular filtration rate. All participants were required to use an insulin pump and a continuous glucose monitor (CGM) to ensure accurate tracking of insulin delivery. Participants then underwent a DEXA scan to quantify body composition and received instructions on study procedures, including diet plans and logging food intake on the MyFitnessPal mobile platform.

#### Nutrition interventions

**Fig. 1:**
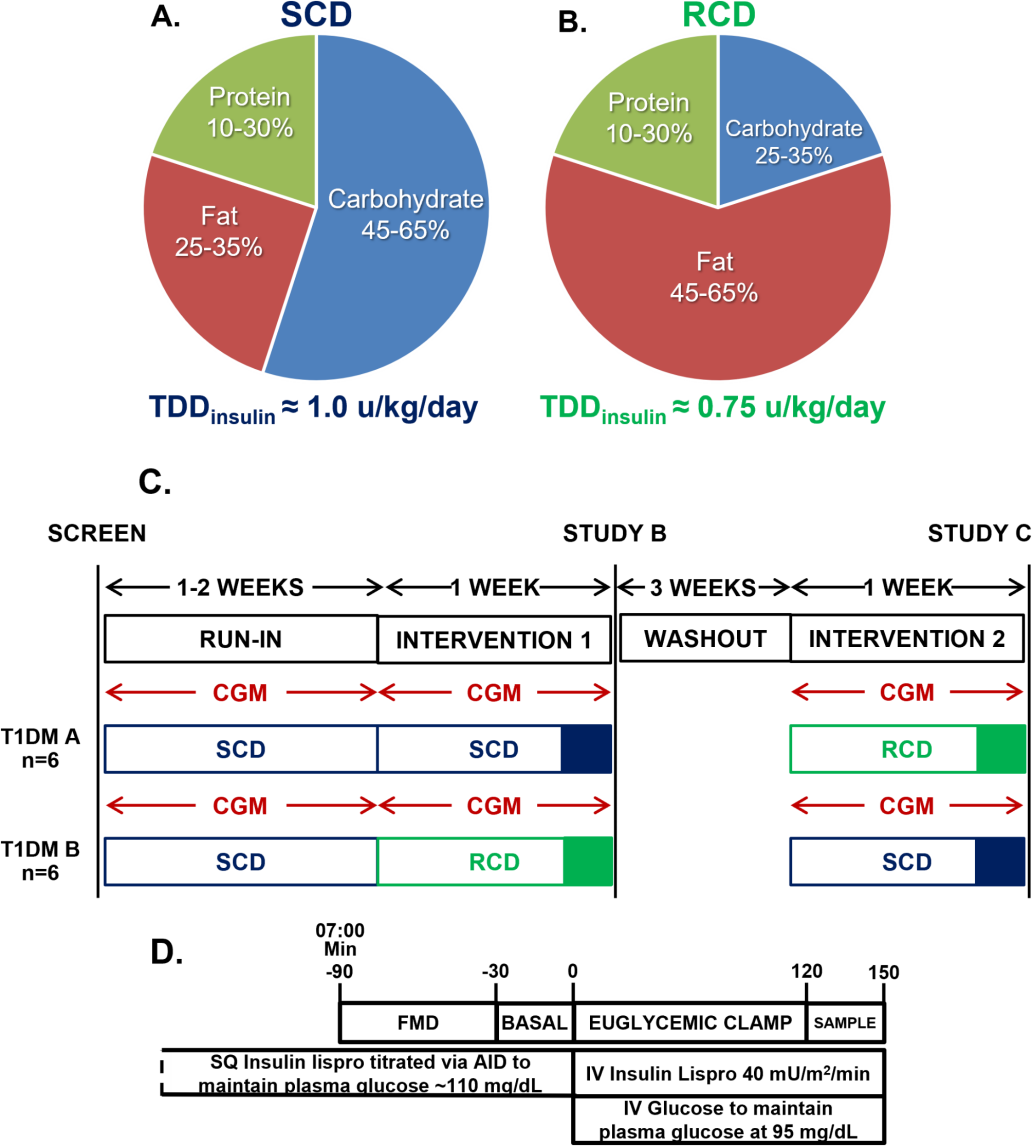
Study design and diet macronutrients. **A** Macronutrient composition of isocaloric, standard carbohydrate diet (SCD) **B** Macronutrient composition of isocaloric reduced carbohydrate diet (RCD). Percentages indicate percent of caloric intake from each macronutrient. The study design anticipated total daily dose of insulin (TDD_insulin_) would be 0.25 units/kg/day lower on RCD than SCD. **C** Schematic of crossover study design. T1DM A = participants undergoing treatment order (**A**) T1DM B = participants undergoing treatment order (**B**) CGM = continuous glucose monitoring. Solid shaded squares at the end of each diet intervention indicates the study team supplied all food in the final 24 h prior to each study. **D** Schematic for research visit procedures. FMD = flow mediated dilation. AID = automated insulin delivery. SQ = subcutaneous

We provided two tailored, isocaloric one-week diet plans calculated via the Mifflin–St. Jeor formula [30], adjusted for activity. The SCD followed recommended macronutrient distributions (45–65% carbohydrate, 10–30% protein, 25–35% fat) [31]. The RCD reduced carbohydrate to 25–35%, raised fat to 45–65%, and maintained protein at 10–30% (Fig. 1A–B). Reducing carbohydrate from ≈ 50% to ≈ 25% of energy intake was expected to halve prandial insulin needs and lower the total daily dose of insulin (TDD_insulin_) by ≈ 25%, a more robust insulin reduction than typically achieved by SGLT2 inhibitors or GLP-1 receptor agonists [25–29]. Thus, this composition aimed to substantially reduce iatrogenic peripheral hyperinsulinemia without resorting to an extremely low-carbohydrate diet, allowing us to test its impact on insulin sensitivity and endothelial function.

Participants prepared their own meals according to assigned diets on days 1–6. On day 7, they consumed standardized, pre-portioned 2,000-kcal/day meals provided by the study team, ensuring controlled macronutrient intake before metabolic testing.

We chose a one-week intervention to optimize adherence, capture the known time course (days to a week) of hyperinsulinemia-induced changes in insulin sensitivity and endothelial function [9–12], and minimize confounding factors like weight or fitness changes.

After a 1–2-week run-in to ensure accurate logging and stable glycemic control using automated insulin delivery, participants were randomised (permuted blocks of four in REDCap) to SCD or RCD first, followed by a three-week washout, a second run-in, and then the alternate diet (Fig. 1C). Completion of nutrition logs and pump data downloads was required for inclusion in correlation analyses; participants missing these data were excluded from those specific analyses.

#### Research visits

Before each visit, participants avoided aspirin and vitamins for three days, NSAIDs for one day, and caffeine, tobacco, and physical activity for 12 h to minimize confounding. Female participants scheduled visits during days 2–10 of their menstrual cycle (follicular phase) to minimize hormonal influences on FMD measurements. The night before, participants consumed a standardized dinner between 5 and 7 PM, then fasted. Overnight, their automated insulin delivery systems targeted a glucose level of ≈ 110 mg/dL (6.11 mmol/L, Fig. 1D). All participants used insulin lispro for 24–48 h pre-study to standardize insulin. At the research center, participants arrived at 7 AM for vital signs and anthropometric measurements and females underwent pregnancy testing.

After resting supine for ten minutes in a controlled environment, we assessed endothelial function by performing a flow-mediated vasodilation (FMD) test. Baseline brachial artery diameter (D_base_) was measured using a Phillips EPIQ 7 C ultrasound with an L12-3 transducer (Bothell, Washington, USA). To induce ischemia, we inflated a sphygmomanometer cuff to 200 mmHg for five minutes. Starting 60 s after cuff deflation, an ultrasonographer measured brachial artery diameter during reactive hyperemia for ten seconds (D_peak_). Brachial artery diameters were independently measured using Brachial Analyzer 5.0 software (Medical Imaging Applications, LLC, Coralville, Iowa, USA). The ultrasonographer and the investigator measuring diameters were blinded to treatment order. The investigator responsible for assigning diets did not measure study outcomes.

After a ten-minute rest, we measured endothelium-independent vasodilation by administering 400 µg of sublingual nitroglycerin and measuring brachial artery diameter changes three minutes later. If systolic blood pressure was below 100 mmHg, we omitted this procedure.

Following vascular function studies, we initiated a hyperinsulinemic, euglycemic clamp to quantify insulin sensitivity. Research staff inserted angiocatheters into a vein in each arm, placing a warmer around the site of one of the angiocathers to arterialize venous blood.

We then conducted a hyperinsulinemic-euglycemic clamp to quantify insulin sensitivity. Catheters were inserted into each arm, with one site warmed to arterialize venous blood. Participants suspended their insulin pumps, and a primed constant intravenous insulin lispro infusion began at 40 mU/m^2^/min, a rate that fully inhibited hepatic glucose production in our previous studies of similar participants [14].

During the 150-minute insulin infusion, we collected arterialized blood samples every 5–10 min to measure plasma glucose using a YSI 2300 analyzer. We adjusted the intravenous infusion rate of a 20% dextrose solution to maintain plasma glucose at 95 ± 3 mg/dL (5.27 ± 0.17 mmol/L). The investigator adjusting the glucose infusion rate (GIR) was blinded to diet assignment. During the final 30 min, we collected additional blood samples for metabolic and hormonal measurements under insulin-stimulated conditions.

After the clamp, participants resumed using their insulin pumps, and the dextrose infusion was gradually reduced. Participants were discharged after lunch.

### Analytical procedures

Arterialized venous blood samples were collected into tubes with potassium EDTA, chilled on ice, centrifuged (15 min at 3,000 RPM, 4 °C), and plasma stored at − 80 °C. Lactate, alanine, and glycerol were measured fluorometrically [32, 33]; NEFAs were quantified by colorimetry (Wako Life Sciences); catecholamines by HPLC [34]; and insulin, glucagon, C-peptide, and cortisol by RIA (MilliporeSigma) [33]. Basal 17β-estradiol was also assayed by RIA (MP Biomedicals). Glucose was measured by the glucose oxidase method (YSI 2300). Cytokine levels were measured using Luminex-based Milliplex Multiplex Panels (MilliporeSigma). Lipoprotein particle concentrations and diameters were measured using NMR spectroscopy (NMR LipoProfile, Labcorp).

### Calculations

Insulin sensitivity, the primary outcome, was quantified using trapezoidal approximation to calculate the area under the curve for GIR during the final 30 min of the clamp, divided by the 30-minute time interval. Thus, we used the average GIR across the last 30 min to represent steady-state insulin action. To express GIR in mg of glucose per kg of fat-free mass per minute (mg/kg FFM/min), we divided the dextrose infusion rate by the fat-free mass determined by DEXA.

Endothelium-dependent FMD, the key secondary outcome, was calculated as the percentage change in brachial artery diameter from baseline:

$$FMD (\%)=\frac{{D}_{peak}-{D}_{base}}{{D}_{base}}.$$

To address potential bias due to D_base_ influencing FMD calculations, we applied allometric scaling (detailed in supplemental appendix). Endothelium-independent vasodilation (NMD) was calculated similarly:

$$NMD (\%)=\frac{{D}_{peak}-{D}_{base}}{{D}_{base}}.$$

Whole-body insulin clearance of peripherally administered insulin was estimated by dividing the intravenous insulin infusion rate by the steady-state plasma insulin concentrations during the final 30 min of the clamp. Steady-state insulin was determined by averaging plasma insulin measurements taken at 15-minute intervals.

To assess cytokine responses to insulin, we calculated the fold-change between insulin-stimulated and basal cytokine concentrations, then log₂-transformed these values to normalize the data and express relative changes on a linear scale.

### Statistics

We initially planned to enroll 20 participants. This target was based on a minimal detectable difference approach for our primary outcome, the GIR during the final 30 min of the clamp. Using an anticipated pooled standard deviation of 0.8 mg/kg/min, based on [11], a two-sided α of 0.05, and 80% power, we calculated that a true difference of ~ 0.53 mg/kg/min in GIR between the two diet interventions would be detectable. After analyzing data from the first ten participants, a preplanned interim analysis using O’Brien-Fleming criteria (p-value threshold of 0.0051) showed a strong trend toward negative results for our key outcomes (GIR and FMD). Given the modest differences observed and the impracticality of achieving statistical significance without an excessively large sample size, we concluded the study after enrolling 14 participants.

Our final analysis used the Wilcoxon signed-rank test to compare GIR and FMD between diets (GraphPad Prism, version 10.3.1). Spearman’s rank-order correlation assessed relationships between GIR, FMD, and metabolic parameters. A repeated-measures ANOVA tested for effects of treatment order on outcomes (IBM SPSS version 29.0.2.0). Data are summarized as medians and interquartile ranges unless otherwise specified.

## Results

### Participant characteristics

Fourteen participants enrolled in the study and completed the screening visit. Two did not proceed past the run-in period due to scheduling conflicts and inconsistent nutrition logging. The remaining 12 participants completed both diet interventions and were included in the analysis. Their clinical characteristics are summarized in Table 1.

**Table 1:** Baseline characteristics of participants. Continuous variables are summarized as medians (interquartile range). Categorical and ordinal variables are expressed as percentages and counts

Baseline characteristic	Participants (*n* = 12)
Male sex, % (n)	42% (5)
Age, years (interquartile range, total range)	33.9 (25.0–39.0, 23.3–49.9)
Weight, kg	75.3 (66.8–92.0)
Height, m	1.75 (1.68–1.88)
BMI, kg/m^2^	26.5 (21.7–28.4)
Waist-to-hip ratio	0.83 (0.75–0.88)
HbA1c, %	7.1 (6.6–7.7)
HbA1c, mmol/mol	54 (49–61)
Type 1 diabetes duration, years	20.5 (15.3–25.0)
Race, % (n)	
White	92% (11)
Black	8% (1)
Systolic blood pressure (mmHg)	120 (118–136)
Diastolic blood pressure (mmHg)	82 (78–90)
Heart rate (bpm)	65 (60–68)
Insulin analog used, % (n)	
Lispro	50% (6)
Aspart	42% (5)
Fast-acting insulin aspart	8% (1)

### Food consumption

During the one-week RCD, carbohydrate intake was 25% lower and fat intake was 35% higher than during the SCD (Table 2). In the 24 h before testing, when participants consumed standardized meals, carbohydrate intake was 51% lower and fat intake was 44% higher with the RCD. Total caloric intake remained similar between diets. Median body weight changed minimally during the study [75.3 kg (IQR 66.8–92.0) at screening vs. 75.8 kg (IQR 66.5–93.3) after RCD vs. 75.6 kg (IQR 67.2–90.8) after the SCD (supplemental Fig. 1)].

**Table 2:** Dietary intake during interventions for the Reduced-Carbohydrate diet (RCD) vs. the Standard‐Carbohydrate diet (SCD). Values represent group medians and interquartile ranges

	RCD (*n* = 8)	SCD (*n* = 7)
7-day mean daily carbohydrate consumption (g/day)	165 (143–201)	222 (201–233)
1-day daily carbohydrate consumption immediately before clamp (g/day)	117 (94–146)	243 (200–281)
7-day mean daily fat consumption (g/day)	92 (69–134)	70 (62–74)
1-day daily fat consumption immediately before clamp (g/day)	91 (65–102)	63 (55–67)
7-day mean daily caloric consumption (kcal/day)	1,805 (1,497-2,698)	1,837 (1,791-2,750)
1-day daily carbohydrate consumption immediately before clamp (kcal/day)	1,830 (1,410-2,050)	1,940 (1,663-2,130)

### Insulin delivery and glycemic control

**Fig. 2:**
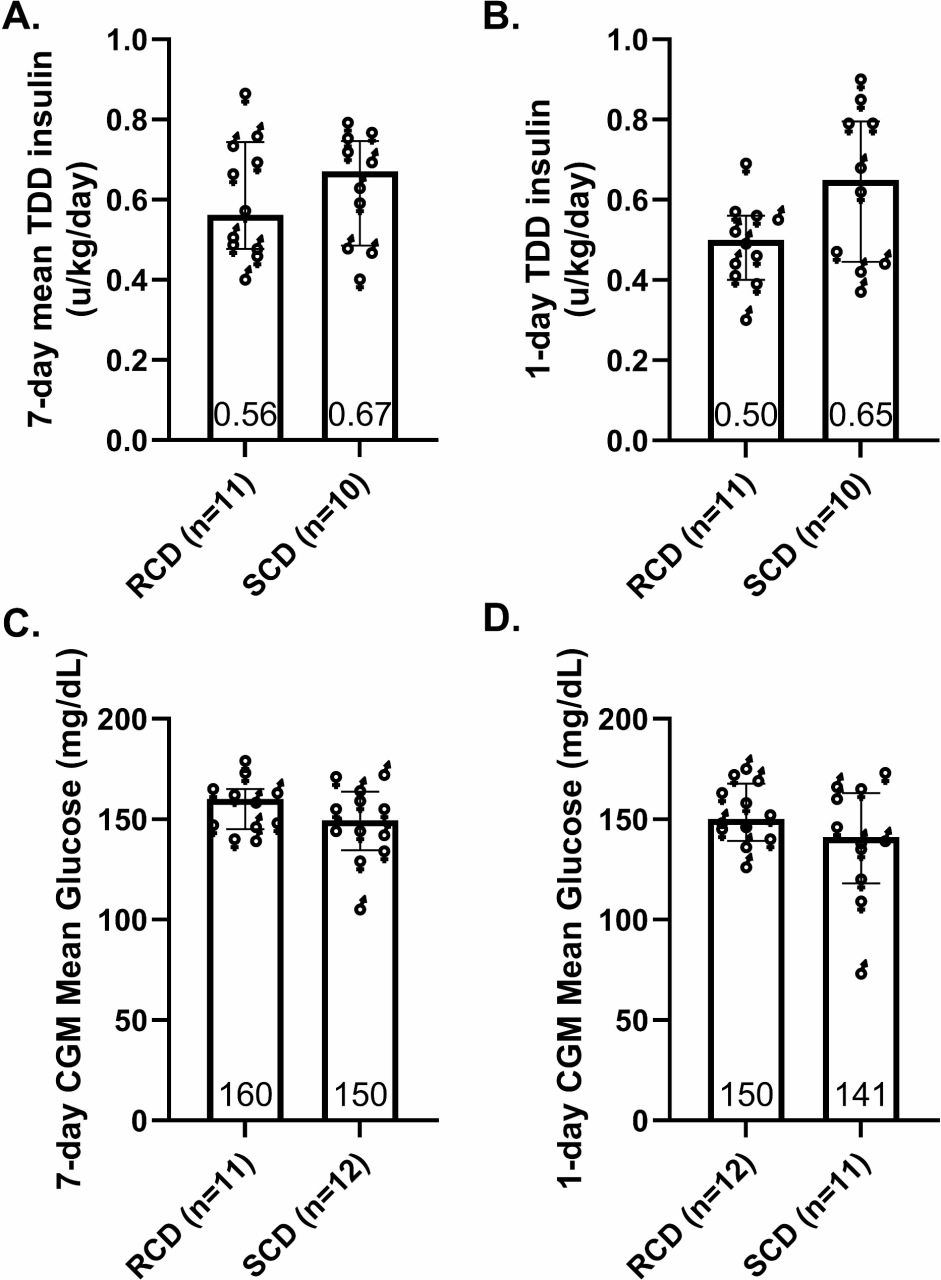
Insulin dose and glycemic control. Total daily dose (TDD) of insulin (**A**-**B**) and continuous glucose monitor (CGM) data (**C**-**D**) during the week and 24 h preceding each experiment. Column scatter plots show the medians of mean daily insulin dose or mean CGM glucose levels during reduced carbohydrate diet (RCD) and standard carbohydrate diet (SCD) interventions, along with the interquartile range

During the week preceding each study, the TDD_insulin_ was 16% lower with the RCD than the SCD (Fig. 2A). In the 24 h before the study, when participants were provided with a standardized diet for each intervention, the TDD_insulin_ was 24% lower (Fig. 2B). Median CGM glucose levels were similar between diets during both periods (Fig. 2C-D).

### Flow mediated dilation

**Fig. 3:**
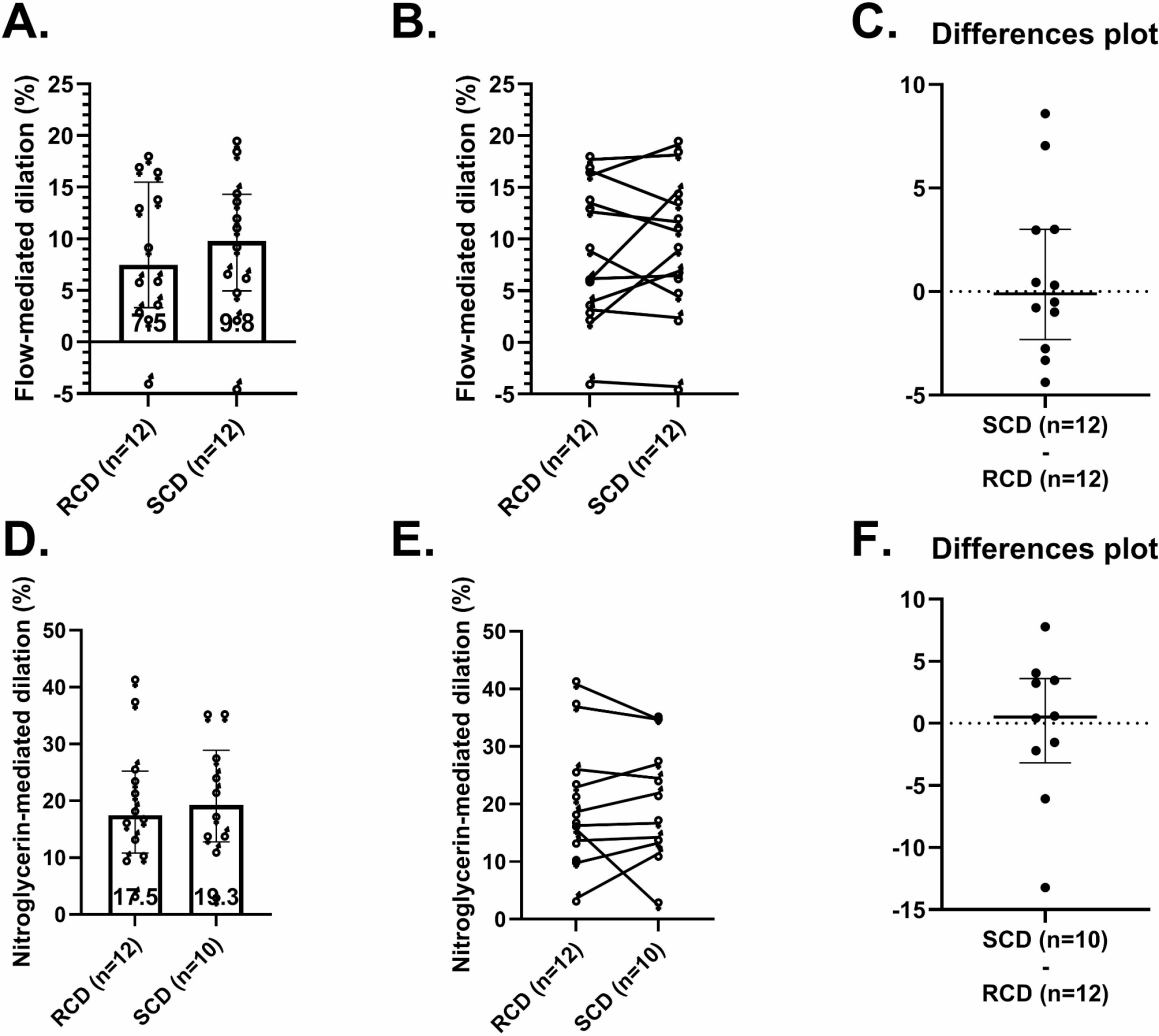
Endothelial function following interventions. Endothelium-dependent flow-mediated dilation (FMD, **A**–**C**) and endothelium-independent nitroglycerin-mediated dilation (NMD, **D**–**F**) after the reduced-carbohydrate diet (RCD) and standard-carbohydrate diet (SCD) interventions. Plots **A** and **D** show individual FMD and NMD data, intervention medians, and the interquartile ranges. Plots B and E show within-participant changes in FMD and NMD between interventions. Plots **C** and **F** show within-participant differences in FMD and NMD between interventions (SCD minus RCD) for each individual, along with median and interquartile ranges for these differences. Per protocol, two participants did not receive nitroglycerin following the SCD because of low systolic blood pressure

Endothelium-dependent FMD was similar between interventions. FMD was 7.50% (IQR 3.25–15.5%) after the RCD and 9.81% (IQR 4.96–14.3%) after the SCD (*p* = 0.91, median difference = 0.10%, 95% CI of differences = -2.76–3.01%, Fig. 3A–C). Endothelium-independent NMD also showed minimal differences between diets (Fig. 3D–F). Allometric scaling did not alter these results (supplemental Fig. 2).

During the RCD, FMD inversely correlated with CGM glucose averages (ρ = -0.645, *p* = 0.032 for the 7-day CGM average and ρ = -0.593, *p* = 0.042 for the 1-day average), indicating that higher glucose levels were associated with poorer endothelial function. No such correlations were found during the SCD (ρ = 0.333, *p* = 0.291 for the 7-day CGM average and ρ = 0.291, *p* = 0.385 for the 1-day average). Additionally, FMD did not correlate with insulin-related parameters or fat intake during either diet, suggesting these factors did not independently influence endothelial function. Treatment order had no significant effect on FMD.

### Baseline lipid profiles

NMR lipoprotein analysis showed no significant differences between the RCD and SCD in lipoprotein particle concentrations or sizes, including VLDL, LDL, HDL, and cholesterol measures such as total cholesterol, LDL-C, HDL-C, non-HDL-C, triglycerides, and ApoB (Supplemental Figs. 3–5).

### Hyperinsulinemic, euglycemic clamp

#### Hormone and glucose concentrations

**Fig. 4:**
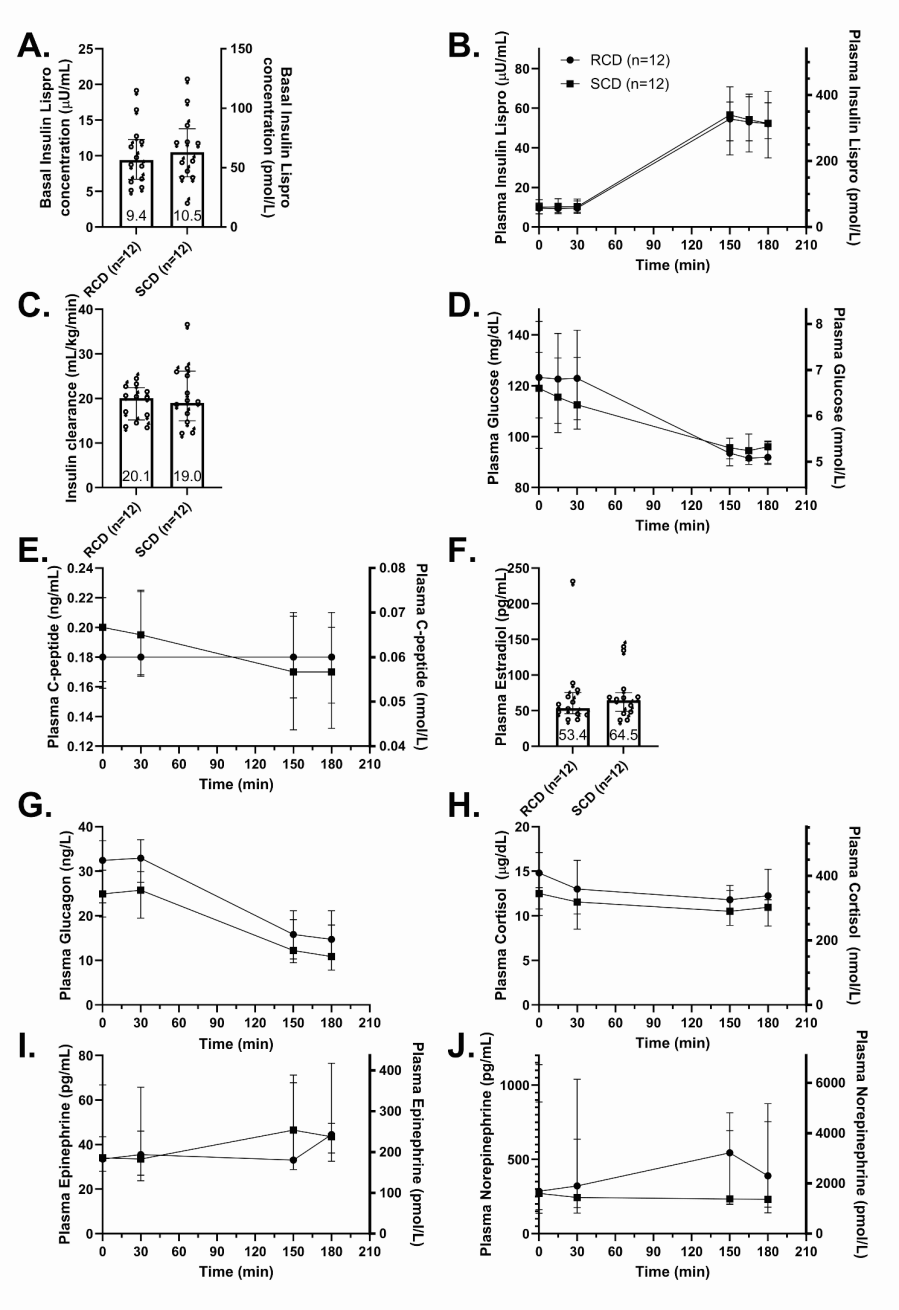
Hormone and glucose parameters. Plasma concentrations of basal insulin lispro (**A**), insulin lispro during the clamp (**B**), glucose (**D**), C-peptide (**E**), basal estradiol (**F**), glucagon (**G**), cortisol (**H**), epinephrine (**I**), and norepinephrine (**J**), as well as insulin clearance (**C**) during hyperinsulinemic, euglycemic clamp studies. Median concentrations are shown as circles for the reduced-carbohydrate diet (RCD) and squares for the standard-carbohydrate diet (SCD). Insulin clearance was calculated by dividing the intravenous insulin infusion rate by steady-state insulin concentrations during the last 30 min of the clamp (**C**). Error bars represent the interquartile range

Basal plasma insulin lispro concentrations were similar between interventions (RCD: 56 pmol/l [IQR 40–74], SCD: 63 pmol/l [IQR 43–83], Fig. 4A) and increased approximately fivefold during the hyperinsulinemic-euglycemic clamp (Fig. 4B). Insulin clearance remained consistent between the two clamp studies (Fig. 4C). Median plasma glucose levels were comparable between diets during both the basal and clamp periods (Fig. 4D). Plasma C-peptide concentrations were low but detectable in both interventions (Fig. 4E). Basal plasma estradiol levels were similar between interventions and comparable to male levels, minimizing potential confounding effects on FMD (Fig. 4F). During the clamp, plasma glucagon levels decreased to about half of basal levels (Fig. 4G). Plasma cortisol, epinephrine, and norepinephrine remained at basal levels throughout the study (Fig. 4H–J).

#### Metabolite response

**Fig. 5:**
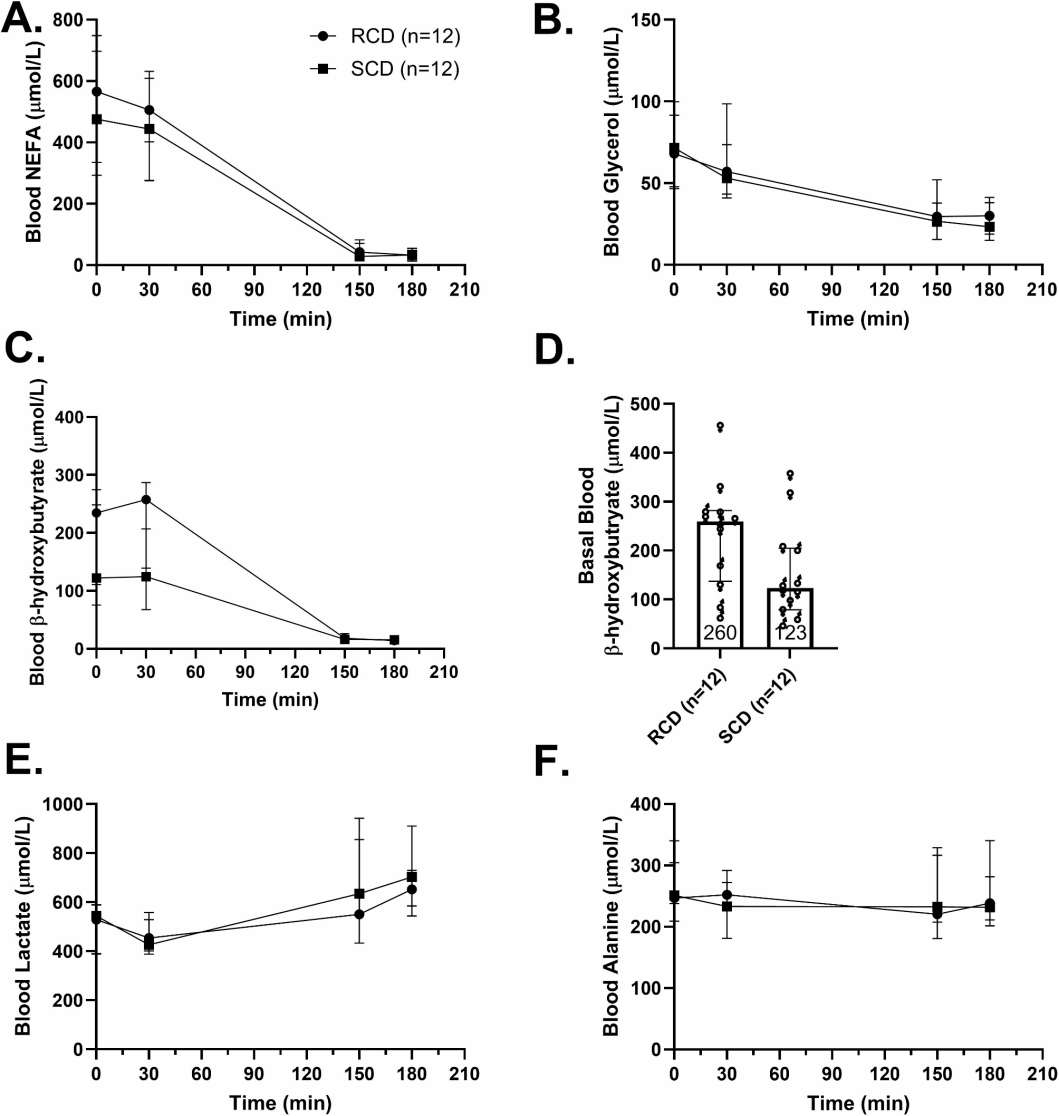
Metabolite responses. Blood concentrations of non-esterified fatty acids (**A**), glycerol (**B**), β-hydroxybutyrate (**C**), β-hydroxybutyrate during the basal period (**D**), lactate (**E**), and alanine (**F**). Median concentrations are shown as circles for reduced carbohydrate diet (RCD) studies and as squares for standard carbohydrate diet (SCD) studies. Error bars represent the interquartile range

Fasting NEFA and glycerol levels were similar between diets (Fig. 5A–B). During the clamp, insulin suppressed lipolysis, reducing NEFA levels to undetectable and halving glycerol levels. Basal β-hydroxybutyrate levels were higher after the RCD (260 µmol/L (IQR 137–282) vs. 123 µmol/L (IQR 78.9–205) after RCD vs. SCD, respectively, median difference 53.9 µmol/L, 95% CI 2.70–147 µmol/L, Fig. 5C-D) but were suppressed to undetectable levels during insulin infusion. Blood lactate levels rose modestly and similarly during the clamp in both diets (Fig. 5E), while alanine levels remained stable (Fig. 5F).

#### Cytokine response

Baseline levels of pro-inflammatory cytokines, soluble endothelial adhesion molecules, and fibrinogen were similar between the RCD and SCD (Supplemental Figs. 6–7). During insulin infusion, the RCD elicited modest increases in IL-1α, with smaller upward trends for IL-1β and IL-6. In contrast, following SCD these cytokine levels were either unchanged or slightly suppressed (Supplemental Fig. 8). Endothelial activation markers remained unchanged in both diets (Supplemental Fig. 9). Fibrinogen levels increased modestly with insulin in both conditions but rose significantly more after the RCD (*p* = 0.009), suggesting a reduced carbohydrate state may amplify insulin-induced fibrinogen responses.

#### Insulin sensitivity

**Fig. 6:**
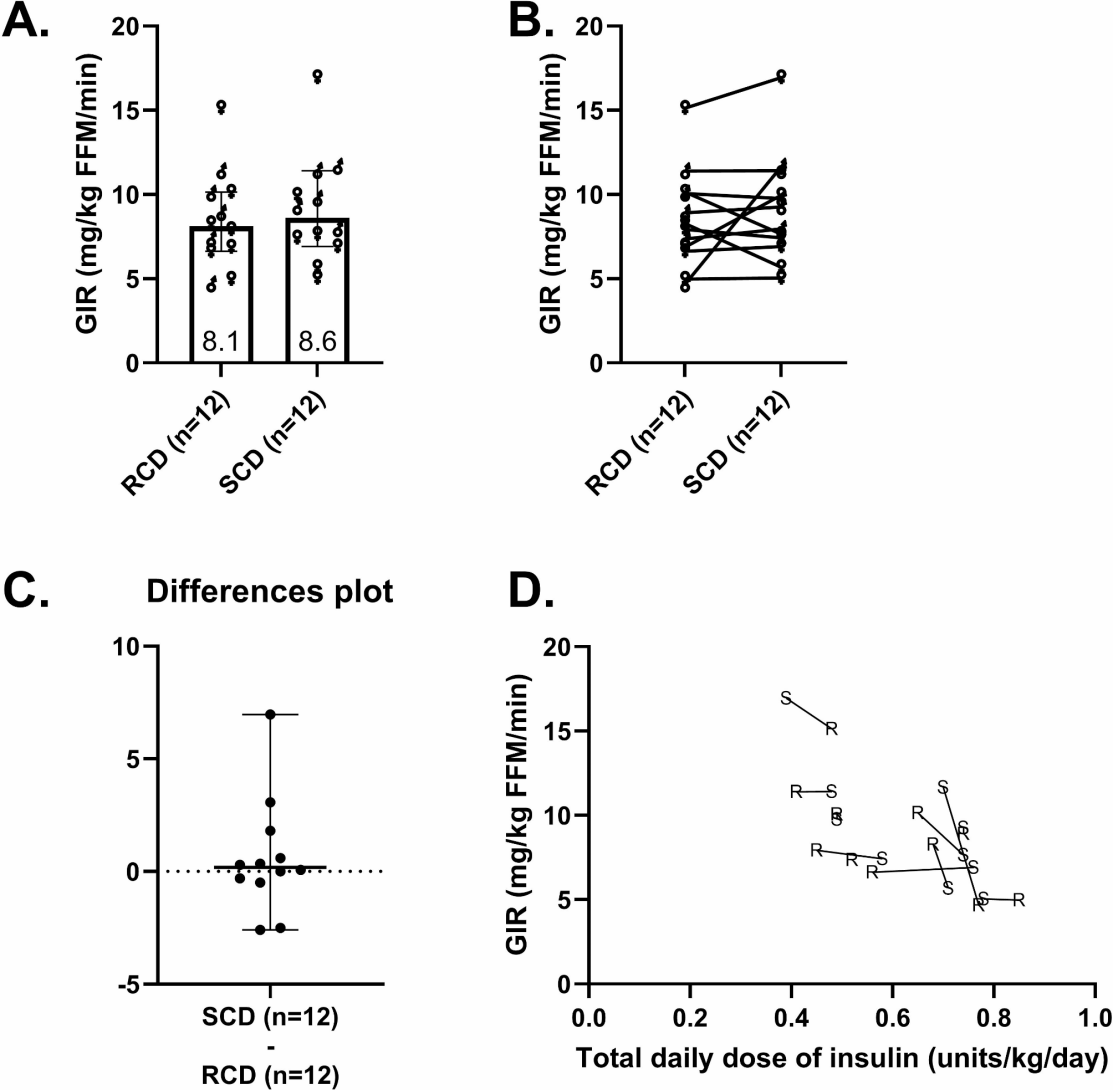
Insulin sensitivity and insulin dose relationship. Glucose infusion rate (GIR) during the final 30 min of hyperinsulinemic, euglycemic clamp studies parameterizing insulin sensitivity following reduced-carbohydrate diet (RCD) and standard-carbohydrate diet (SCD) interventions. Plot **A** shows individual GIR data, intervention medians, and the interquartile ranges. Plot **B** shows within-participant changes in GIR between interventions. Plot **C** shows within-participant changes in GIR between interventions (SCD minus RCD) for each individual, along with median and interquartile ranges for these differences. Plot **D** shows the relationship between the total daily dose of insulin (averaged over the seven days of each intervention) and GIR during the RCD and SCD interventions. Points marked “R” represent the RCD study and points marked “S” represent the SCD study, with lines connecting each participant’s two studies

Insulin sensitivity, the primary outcome measured by the GIR during the last 30 min of the hyperinsulinemic-euglycemic clamp, showed no significant difference between diets. GIR was 8.1 mg/kg FFM/min (IQR 6.7–10.1) after the RCD and 8.6 mg/kg FFM/min (IQR 7.0–11.0) after the SCD (*p* = 0.47, median difference = 0.2 mg/kg FFM/min, 95% CI of differences = -0.5 to 1.8, Fig. 6A-C).

GIR demonstrated a significant inverse correlation with TDD_insulin_ during both interventions. The correlation was ρ = − 0.609 (*p* = 0.047) for the RCD and stronger at ρ = − 0.782 (*p* = 0.008) for the SCD. This suggests that individuals with higher insulin doses had lower insulin sensitivity in both interventions (Fig. 6D). No significant correlations were found between GIR and glycemic parameters (e.g., 1-day or 7-day CGM averages) during either intervention, suggesting that glycemic control was not directly linked to insulin sensitivity in this study. Additionally, the inverse correlation between GIR and basal insulin levels approached statistical significance during both interventions (RCD: ρ = -0.538, *p* = 0.071; SCD: ρ = -0.501, *p* = 0.097), implying a trend where higher basal insulin levels are associated with reduced insulin sensitivity.

Fat intake did not correlate with GIR during either diet, indicating that higher fat consumption with the RCD did not affect insulin sensitivity. Treatment order also had no significant effect on GIR.

## Discussion

Our trial suggests that although an RCD reduces TDD_insulin_ by 16% type 1 diabetes, it does not improve insulin sensitivity or endothelial function compared to an SCD. Our hypothesis that the RCD would reduce iatrogenic peripheral hyperinsulinemia, thereby enhancing insulin sensitivity and endothelial function, was not supported by the data. In this single-blinded, random-order crossover study, we compared a one-week RCD to an isocaloric SCD. The primary outcomes—insulin sensitivity, measured by the GIR during a hyperinsulinemic, euglycemic clamp, and endothelial function, assessed by FMD—showed no significant differences between the two interventions.

Our hypothesis was grounded in robust evidence that sustained hyperinsulinemia at levels typical in type 1 diabetes can induce insulin resistance and impair endothelial function. Marangou et al., Rizza et al., and Del Prato et al. provided compelling evidence that sustained hyperinsulinemia for periods ranging from 20 h to 4 days leads to insulin resistance in healthy humans [9–11]. Similarly, Campia et al. and Arcaro et al. showed that 4 to 6-hour hyperinsulinemic infusions markedly reduced endothelium-dependent vasodilation [12, 35]. These studies led us to hypothesize that reducing insulin exposure through an RCD, even for one week, would improve insulin sensitivity and endothelial function in type 1 diabetes.

While prior research shows sustained hyperinsulinemia can induce insulin resistance [9–11] and endothelial dysfunction [12, 35], we observed no significant improvements with the RCD. Notably, fasting β-hydroxybutyrate levels were higher after the RCD than after the SCD, consistent with the expected metabolic impact of carbohydrate reduction. However, our participants’ automated insulin delivery systems delivered basal insulin similarly during the overnight fasting period before each test. This consistency may have diminished the effect of any differences in insulin exposure between the RCD and SCD by the time of our measurements the next morning, effectively “washing out” any potential benefits of the RCD. In contrast, previous studies that induced hyperinsulinemia measured insulin sensitivity [9–11] and endothelial dysfunction [12, 35] immediately after the intervention. These findings suggest that the effect of sustained physiologic hyperinsulinemia on insulin sensitivity and endothelial function may be more dynamic than previously appreciated, with rapid, short-term fluctuations potentially playing a role.

The significant inverse correlations between TDD_insulin_ and GIR across both interventions support the notion that individuals requiring higher insulin doses were more insulin resistant. Although the RCD reduced TDD insulin by 16%, this reduction did not translate into lower fasting insulin levels, likely because the overnight automated insulin delivery maintained comparable basal insulin exposure with both interventions. We speculate that measuring daytime postprandial insulin concentrations might have revealed differences corresponding to the reduced prandial insulin dosing, and that such differences could better reflect the impact of carbohydrate reduction on circulating insulin levels. This observation underscores the complexity of relationship between insulin dosing and insulin sensitivity in type 1 diabetes. While reducing TDD_insulin_ without worsening glycemia remains valuable, our findings suggest timing of insulin delivery may also play an important role. Our future studies will clarify the dynamic relationship between iatrogenic hyperinsulinemia, insulin sensitivity, and endothelial function in type 1 diabetes.

Although insulin sensitivity and endothelial function did not differ significantly between the interventions, we observed modest but unexpected differences in the inflammatory response to insulin. Specifically, the RCD clamp showed an increase in IL-1α and smaller trends in IL-1β and IL-6, despite two factors—the modestly higher levels of β-hydroxybutyrate and the insulin infusion—that would typically temper inflammation. These findings were surprising given prior evidence that both β-hydroxybutyrate [36–38] and insulin [39–43] can exert anti-inflammatory effects in several conditions, suggesting that a reduced carbohydrate state in type 1 diabetes may alter insulin’s influence on cytokine production. By contrast, the SCD clamp did not appear to raise inflammatory cytokines. Although these results are intriguing, further investigation is needed, especially since our study was not primarily designed or powered to explore the interaction between diet, β-hydroxybutyrate, and cytokine responses to insulin.

This study has several strengths. First, the randomized, crossover design allowed each participant to serve as their own control, minimizing the impact of inter-individual variability on study outcomes. Second, the study was conducted in a “free-living” environment, enhancing its external validity and relevance to real-world settings, as participants adhered to dietary interventions while managing their diabetes with automated insulin delivery systems. Third, the use of automated insulin delivery ensured precise control of insulin dosing, providing an accurate measure of insulin exposure during both interventions. Fourth, we employed robust, well-validated techniques to measure key outcomes, such as insulin sensitivity (using hyperinsulinemic, euglycemic clamps) and endothelial function (using FMD). Fifth, using standardized meals before metabolic testing ensured consistency in macronutrient intake in the critical 24-hour window before the clamp and vascular function tests, limiting potential confounding from dietary intake. Lastly, to our knowledge this is the first randomized controlled trial to compare the effects of a reduced-carbohydrate diet with a standard-carbohydrate diet on insulin sensitivity and endothelial function in individuals with type 1 diabetes, providing valuable insights into the cardiometabolic impact of dietary interventions.

This study also has some limitations. First, while the sample size was sufficient to detect differences in insulin sensitivity and endothelial function, it was limited for more granular analyses, such as correlations and multivariable linear regression, which may have missed subtle effects. Second, the single-blinded design, where participants were aware of their nutritional intake, could have introduced some behavioral bias, influencing adherence or lifestyle choices outside of the controlled setting. Third, although participants were provided with standardized meals the day before testing, dietary compliance during the remainder of the study week relied on participants preparing their own meals. This reliance on self-reporting and meal preparation may have influenced the consistency of the intervention, which along with incomplete dietary logging by some participants, could have influenced study outcomes. Fourth, due to limitations in our insulin pump-download software, we were unable to isolate overnight basal insulin delivery separately from total daily insulin dose. Fifth, while our measurement of conduit artery FMD is an established marker of endothelial function, we cannot exclude the possibility that insulin-mediated effects on microvascular function might differ, with microvessels potentially responding to hyperinsulinemia in ways not captured by conduit artery FMD. Finally, while the “free-living” nature of the study reflects real-world conditions, it also introduces variability in diet and behavior that could not be fully controlled, potentially introducing unmeasured confounding variables.

## Conclusions

In conclusion, our findings suggest that although an RCD significantly reduces the total daily dose of insulin in individuals with type 1 diabetes, it does not lead to measurable improvements in insulin sensitivity or endothelial function compared to an SCD. These results underscore the complex and dynamic relationship between insulin exposure, insulin sensitivity, and endothelial function in type 1 diabetes. Our future research will determine how a short-term reduction in iatrogenic peripheral hyperinsulinemia improves insulin sensitivity and endothelial function, aiming to uncover novel strategies for mitigating cardiometabolic risk in this population.

## Supplementary Information

Below is the link to the electronic supplementary material.

Supplementary Material 1

## Data Availability

Data is provided within the manuscript or supplementary information files. The datasets generated or analyzed during this study are available from the corresponding author upon reasonable request. The full trial protocol is also available upon reasonable request. No applicable resources were generated.

## Abbreviations

CGM: Continuous glucose monitor
FFM: Fat-free mass
FMD: Flow-mediated vasodilation
GLP-1: Glucagon-like peptide-1
IQR: Interquartile range
RCD: Reduced-carbohydrate diet
SCD: Standard-carbohydrate diet
TDD_insulin_: Total daily dose of insulin
