# Supplementary material for: A one-week reduced-carbohydrate diet to mitigate iatrogenic peripheral hyperinsulinemia does not improve insulin sensitivity or endothelial function in a randomized, crossover trial in patients with type 1 diabetes
Source: Cardiovasc Diabetol. 2025 Mar 5;24:107. doi: 10.1186/s12933-025-02658-z (PMC11884211; doi:10.1186/s12933-025-02658-z)
Supplement: Supplementary file 1 — Supplementary Material 1 [file 12933_2025_2658_MOESM1_ESM.docx]

# SUPPLEMENTAL MATERIAL

1. Supplemental Description of Methods
2. Supplemental Tables
3. Supplemental Figures

# SUPPLEMENTAL DESCRIPTION OF METHODS

## Allometric scaling of FMD data.

We conducted allometric scaling analysis to account for differences in baseline artery diameters, which can bias traditional FMD calculations. By normalizing the peak diameters using a study-population-specific allometric exponent, we provide a more accurate measure of endothelial function that is less influenced by the initial size of the artery. This adjustment ensures that the FMD values reflect true physiological changes rather than size-related artifacts, enhancing the reliability and comparability of our results across different interventions.

We conducted allometric scaling for FMD data using two calculations based on reports by Atkinson and Batterham(1) and McLay and colleagues(2). We first log-transformed both the baseline (D_base_) and peak (D_peak_) brachial artery diameters for each participant following the reduced-carbohydrate diet (RCD) and standard carbohydrate diet (SCD) interventions. Using the Microsoft Excel’s Data Analysis Toolpak, we performed linear regression on the log-transformed data, with ln(D_base_) as the independent variable and ln(D_peak_) as the dependent variable, to obtain the allometric exponent (slope, b) for each intervention.

For the first adjusted FMD calculation we used the formula,

$${Adjusted FMD(\%)}_{1}=\frac{D_{peak}}{D_{base}^{b}}$$

Because first adjusted FMD calculation yields values well in excess of the “standard formula” for FMD (described in the methods section of the manuscript), we also used a second formula, described by McLay and colleagues(2):

$${Adjusted FMD(\%)}_{2}=\frac{D_{peak}-D_{base}}{D_{base}^{b}}$$

Our analyses of allometrically scaled data are described below under supplemental figure 1.

# SUPPLEMENTAL TABLES:


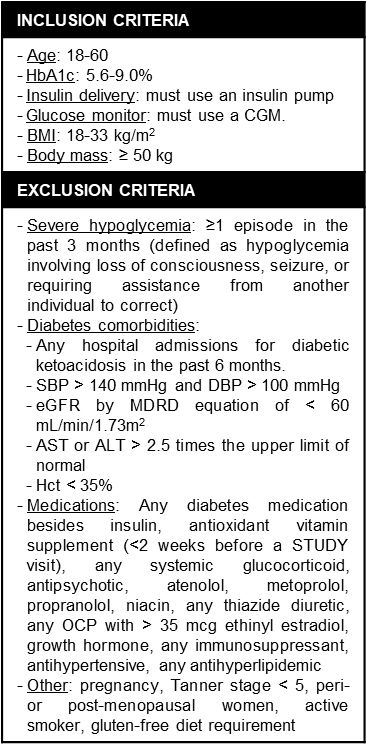


Supplemental Table 1. Inclusion and exclusion criteria. SBP = systolic blood pressure. DBP = diastolic blood pressure. eGFR = estimated glomerular filtration rate. MDRD = modification of diet in renal disease. AST = aspartate aminotransferase. ALT = alanine aminotransferase. Hct = hematocrit. OCP = oral contraceptive pill.

# SUPPLEMENTAL FIGURES:

Supplemental Figure 1: *Individual participant body weights at screening, after reduced carbohydrate diet (RCD), and after standard carbohydrate diet (SCD) interventions.*

Supplemental Figure 2. *Endothelium-dependent flow-mediated dilation data using two different allometric scaling approaches, as described in the supplemental description of methods section. In figures A-C, we conducted allometric scaling using the formula FMD(%) = D_peak_ / D^b^_base_, - 1. In figures D-F, we used the formula FMD(%) = (D_peak_ - D_base_) / D^b^_base_. Plots A and D show individual FMD and NMD data, intervention medians, and the interquartile range. Plots B and E show within-participant changes in FMD and NMD between interventions. Plots C and F show within-participant differences for FMD and NMD between interventions (SCD minus RCD) for each individual, along with median and interquartile range for these differences.*

Supplemental Figure 3. *Median plasma concentrations of lipoprotein particles across interventions: Total VLDL and chylomicron particles (A), large VLDL and chylomicron particles (B), medium VLDL particles (C), small VLDL particles (D), LDL particles (E), large LDL particles (F), small LDL particles (G), IDL particles (H), HDL particles (I), large HDL particles (J), medium HDL particles (K), and small HDL particles (L). Plots show individual participant data, intervention medians, and the interquartile range.*

Supplemental Figure 4. *Median particle sizes and lipoprotein insulin resistance (LP-IR) score across interventions: VLDL particle size (A), HDL particle size (B), LDL particle size (C), and LP-IR score (D). Plots show individual participant data, intervention medians, and the interquartile range.*

Supplemental Figure 5. *Median cholesterol and lipoprotein plasma concentrations across interventions: total cholesterol (A), HDL cholesterol (B), triglycerides (C), ApoB (D), LDL cholesterol (E), VLDL cholesterol (F), and non-HDL cholesterol (G). Plots show individual participant data, intervention medians, and the interquartile range.*

Supplemental Figure 6. *Baseline plasma concentrations of pro-inflammatory cytokines: IL-1α (A-C), IL-1β (D-F), IL-6 (G-I), and TNF-α (J-L). Panels A, D, G, and J display individual cytokine concentrations for each intervention (RCD and SCD), along with medians and interquartile ranges. Panels B, E, H, and K depict within-participant changes in cytokine levels between interventions. Panels C, F, I, and L illustrate the differences in cytokine concentrations (SCD minus RCD) for each participant, with medians and interquartile ranges of these differences.*

Supplemental Figure 7. *Baseline plasma concentrations of cytokines, soluble endothelial activation markers, and acute phase reactants: VEGF-A (A-C), sE-Selectin (D-F), sICAM-1 (G-I), sVCAM-1 (J-L), and fibrinogen (M-O). Panels A, D, G, J, and M display individual data for each inflammatory marker during the RCD and SCD interventions, with medians and interquartile ranges. Panels B, E, H, K, and N show within-participant changes in inflammatory marker levels between interventions. Panels C, F, I, L, and O illustrate differences in inflammatory marker concentrations (SCD minus RCD) for each participant, with medians and interquartile ranges of the differences.*

Supplemental Figure 8. *Log2 fold changes in plasma cytokine levels during insulin infusion relative to baseline (insulin-stimulated / basal insulin) for IL-1α (A-C), IL-1β (D-F), IL-6 (G-I), and TNF-α (J-L). Panels A, D, G, and J show individual participant data for each intervention (RCD and SCD), with medians and interquartile ranges of the fold changes. Panels B, E, H, and K illustrate within-participant changes in cytokine fold responses between interventions. Panels C, F, I, and L display differences in the log2 fold changes (SCD minus RCD) for each cytokine, with medians and interquartile ranges of the differences.*

Supplemental Figure 9. *Log2 fold changes in plasma levels of soluble endothelial activation markers and acute phase reactants during insulin infusion relative to baseline (insulin-stimulated / basal insulin) for VEGF-A (A-C), sE-Selectin (D-F), sICAM-1 (G-I), sVCAM-1 (J-L), and fibrinogen (M-O). Panels A, D, G, J, and M present individual participant data for each intervention (RCD and SCD), with medians and interquartile ranges of the fold changes. Panels B, E, H, K, and N depict within-participant changes in fold responses between interventions. Panels C, F, I, L, and O display differences in the log2 fold changes (SCD minus RCD) for each inflammatory marker, with medians and interquartile ranges of the differences.*

# REFERENCES

1. Atkinson G, Batterham AM: The percentage flow-mediated dilation index: a large-sample investigation of its appropriateness, potential for bias and causal nexus in vascular medicine. Vasc Med 2013;18:354-365

2. McLay KM, Nederveen JP, Koval JJ, Paterson DH, Murias JM: Allometric scaling of flow-mediated dilation: is it always helpful? Clin Physiol Funct Imaging 2018;38:663-669
